# Multi-omics integration for both single-cell and spatially resolved data based on dual-path graph attention auto-encoder

**DOI:** 10.1093/bib/bbae450

**Published:** 2024-09-18

**Authors:** Tongxuan Lv, Yong Zhang, Junlin Liu, Qiang Kang, Lin Liu

**Affiliations:** BGI Research, No. 9, Yunhua Road, Yantian District, Shenzhen 518083, China; College of Life Sciences, University of Chinese Academy of Sciences, No. 19, Yuquan Road, Shijingshan District, Beijing 100049, China; BGI Research, No. 9, Yunhua Road, Yantian District, Shenzhen 518083, China; BGI Research, No. 9, Yunhua Road, Yantian District, Shenzhen 518083, China; BGI Research, No. 9, Yunhua Road, Yantian District, Shenzhen 518083, China; BGI Research, No. 9, Yunhua Road, Yantian District, Shenzhen 518083, China

**Keywords:** multi-omics integration, multi-omics joint analysis, single-cell omics, spatial omics, graph attention auto-encoder

## Abstract

Single-cell multi-omics integration enables joint analysis at the single-cell level of resolution to provide more accurate understanding of complex biological systems, while spatial multi-omics integration is benefit to the exploration of cell spatial heterogeneity to facilitate more comprehensive downstream analyses. Existing methods are mainly designed for single-cell multi-omics data with little consideration of spatial information and still have room for performance improvement. A reliable multi-omics integration method designed for both single-cell and spatially resolved data is necessary and significant. We propose a multi-omics integration method based on dual-path graph attention auto-encoder (SSGATE). It can construct the neighborhood graphs based on single-cell expression profiles or spatial coordinates, enabling it to process single-cell data and utilize spatial information from spatially resolved data. It can also perform self-supervised learning for integration through the graph attention auto-encoders from two paths. SSGATE is applied to integration of transcriptomics and proteomics, including single-cell and spatially resolved data of various tissues from different sequencing technologies. SSGATE shows better performance and stronger robustness than competitive methods and facilitates downstream analysis.

## Introduction

Multi-omics typically includes genomics, transcriptomics, proteomics, metabolomics, epigenomics, phenomics, and other single-omics [1, 2]. Research in these fields has enhanced our understanding of complex biological systems. Multi-omics integration refers to feature representation and dimensionality reduction of different single-omics data to obtain their joint low-dimensional representation [3]. It can provide more comprehensive information for joint downstream analysis. One example is the integration of proteomics and metabolomics, which reveals the regulation of metabolites by proteins and facilitates the prediction and verification of gene functions [4]. In addition, the integration of transcriptomics and DNA methylation makes significant progress in biomedical classification tasks, offering new insights into the understanding and treatment of diseases [5].

The integration of transcriptomics and proteomics is currently one of the focal points [6, 7]. With the continuous development of biology and biomedicine, it has become feasible to collect a large amount of single-omics data. The emergence of spatially resolved transcriptomics and spatially resolved proteomics technologies provides access to the single-omics data with spatial information [8]. In particular, advanced sequencing technologies, such as SPOTS [9], spatial-CITE-seq [10], and Stereo-CITE-seq [11], enable the simultaneous acquisition of transcriptome and proteome data on the same tissue section, thereby better maintaining the homology of different single-omics data. To utilize existing data in joint analysis and facilitate new discoveries in gene regulation, biological evolution, disease treatment, etc., multi-omics integration methods for transcriptomics and proteomics have become effective and important.

While multi-omics integration plays a critical role in advancing research, it faces the challenge of addressing the inherent differences among various single-omics data. Sometimes these differences can be significant, requiring efforts to ensure data consistency in terms of formats, dimensions, units, and normalizations [12]. Different single-omics data can be presented using various methods and tools to convey their biological significance. However, a challenge is that the integrated data still require reliable biological interpretation approaches [13].

Traditional integration methods for transcriptomics and proteomics meet these challenges from statistics, network-based technologies, and other perspectives. MOFA is a typical statistical method in an unsupervised fashion [14]. It infers a low-dimensional representation in terms of interpretable factors that capture the global sources of variation across modalities, helping to identify continuous molecular gradients and discrete sample subgroups. MOFA+, an extension of MOFA, incorporates priors for flexible structure regularization to enable joint modeling of multiple groups and data modalities [15]. Seurat v5 is a network-based method, and it constructs a weighted nearest neighbor graph for data representation by connecting cells that share similarities across modalities, using learned cell-specific weights that determine the relative importance of different single-omics data [16]. Other traditional methods include SCIM [17], GRMEC-SC [18], and Mowgli [19]. Although these methods have made significant contributions, they are generally suitable for scenarios where the nonlinear relationships between transcriptomic and proteomic data are not complex, which is often not the case in real situations.

With the continuous advancement of computing technology, multi-omics integration methods for transcriptomics and proteomics based on deep learning are emerging. TotalVI utilizes a modeling strategy similar to scVI [20] to learn a joint probabilistic representation of paired gene expression and protein data [21]. ScMM addresses the complexity of multimodal single-cell data using a mixture-of-experts multimodal variational auto-encoder [22]. Other deep learning–based methods include scMDC [23], BABEL [24], and InClust+ [25]. These methods partially address the limitations of traditional methods, but there is room for improvement in their performance, and the challenges in multi-omics integration still remain. Additionally, almost all methods are designed for single-cell multi-omics data. When applied to spatial multi-omics data, they fail to incorporate spatial information. The only method we have searched that involves spatial information is SpatialGlue [26]. However, it currently only supports the integration of spatial multi-omics data and cannot be applied to single-cell multi-omics data.

**Table 1:** Details of multi-omics datasets (transcriptomics and proteomics).

Type	Name	Tissue	Technology	*Nc*	*NR*	*Np*
Single-cell	BMNC	Human bone marrow	CITE-seq	30 672	17 009	25
	SLN111_D1	Mouse spleen	CITE-seq	9264	13 553	112
Spatial	SCS_MT	Mouse thymus	Stereo-CITE-seq	20 125	26 439	216

*Nc* represents the number of cells, *NR* represents the number of RNAs, and *Np* represents the number of proteins.

Single-cell multi-omics data have expression profiles with resolution at single-cell level that their integration enables more accurate understanding of complex biological systems. Spatial multi-omics data include expression profiles and spatial information. Spatial transcriptome expression profile is generally sparse, and spatial proteome expression profile is usually less dimensional. However, the introduction of spatial information makes their joint analysis useful for exploring spatial heterogeneity and conducting more comprehensive downstream analysis [27]. Therefore, it is necessary and significant to design a reliable multi-omics integration method for both single-cell and spatially resolved data.

In addition to learning the representation of each single-omics data and obtaining an effective joint representation, multi-omics integration also needs to retain the unique characteristics of each single-omics data. The graph attention auto-encoder (GATE) combines the graph attention mechanism with the auto-encoder [28], which has been verified to efficiently extract low-dimensional representations from complex graph-structured data [29]. This is crucial for multi-omics integration where initial data can be high-dimensional and sparse. The attention mechanism allows the model to selectively focus on important nodes and edges in the input graph, automatically learning which parts are critical for specific tasks [30]. This is particularly advantageous in extracting important biological features from graph-structured data. As a self-supervised learning method, the auto-encoder framework is favorable for multi-omics data without ground truth labels [29]. Specifically, the graph neural network structure integrates information from neighboring nodes in the data through efficient calculations [31, 32]. Notably, existing multi-omics integration methods often focus on shared information among different single-omics data, neglecting the specific information of each single-omics data. The dual-modality factor model can identify and extract shared information across modalities and complementary information specific to each modality [33], which provides guidance for designing a model with a dual-path framework to integrate multi-omics data.

This study presents a multi-omics integration method based on dual-path GATE (SSGATE) for both single-cell and spatially resolved data. For single-cell multi-omics data, it constructs neighborhood graphs based on single-cell expression profiles, while for spatial multi-omics data, it constructs neighborhood graphs based on spatial coordinates. Two single-omics data are input into two GATEs via two separate paths. Two embeddings obtained through the encoders are integrated. Then, they are used for reconstruction separately by the decoders. To train the model more effectively, a combined weighted loss of self-supervision and self-reconstruction losses [34] is adopted. SSGATE is applied to the multi-omics integration of transcriptomics and proteomics, including single-cell and spatially resolved data of various tissues from different sequencing technologies. Benchmarking results verify that SSGATE outperforms competitive methods in terms of performance and robustness. Additionally, SSGATE facilitates downstream analysis, such as cell clustering and developmental trajectory inference.

## Materials and methods

### Datasets and preprocessing

The single-cell multi-omics datasets BMNC [35] and SLN111_D1 [21] and the spatial multi-omics dataset SCS_MT [11] are downloaded (Table 1). They are converted to “h5ad” and “rds” formats for preprocessing and experiments. For transcriptome expression profiles, count depth scaling with subsequent log plus one transformation is used for normalization, and then, the top highly variable genes are selected to reduce the dimensions [36]. For proteome expression profiles, centered log-ratio transformation is used for normalization [37].

**Figure 1:**
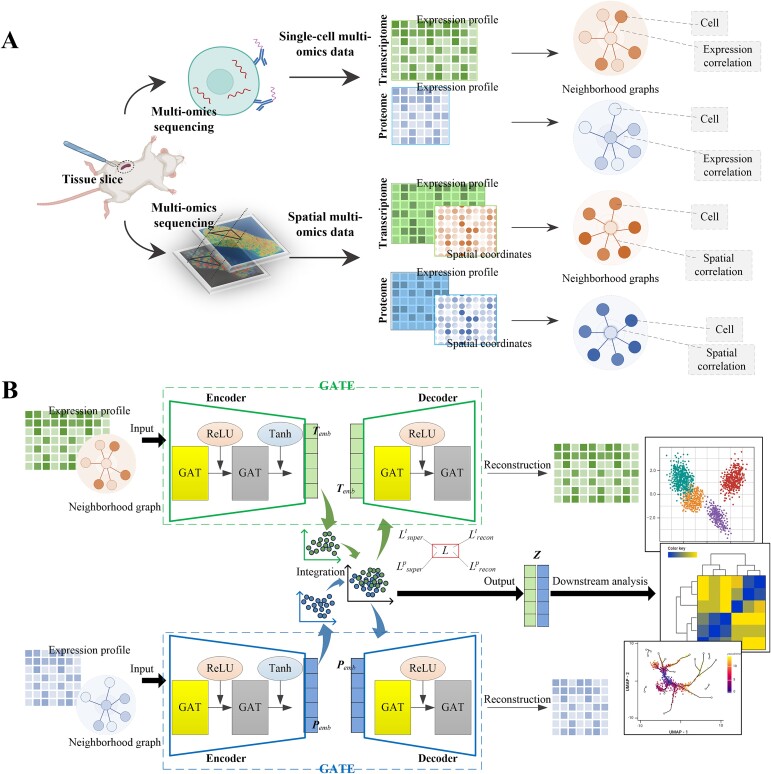
Overview of SSGATE. (**A**) Neighborhood graph construction for single-cell multi-omics and spatial multi-omics data. (**B**) Dual-path graph attention auto-encoder for multi-omics integration. “GATE” represents the graph attention auto-encoder. “GAT“ represents the graph attention layer. “ReLU” and “Tanh” are the activation functions. ***T**_emb_* and ***P**_emb_* are the transcriptome embedding and proteome embedding, respectively. ***Z*** is the joint representation. *L* is the combined weighted loss. *L^t^_recon_* and *L^p^_recon_* are the transcriptome and proteome self-reconstruction losses, respectively. *L^t^_super_* and *L^p^_super_* are the transcriptome and proteome self-supervision losses, respectively.

### Dual-path graph attention auto-encoder for multi-omics integration of both single-cell and spatially resolved data

The overview of SSGATE is shown in Fig. 1. Advanced sequencing technologies provide single-cell multi-omics and spatial multi-omics data. Neighborhood graphs are constructed for transcriptome and proteome data, respectively (Fig. 1A). Two single-omics data are input into two separate GATEs for training through self-supervised learning, and finally, the integrated data are output for downstream analysis (Fig. 1B).

#### Neighborhood graph construction

For single-cell multi-omics data, the neighborhood graphs are constructed based on single-cell expression profiles, where each node represents a cell and each edge represents the expression correlation between two cells. The first step is the neighbor set generation. Due to the high dimensionality of transcriptome expression profile, principal component analysis (PCA) [38] is used to reduce its dimensions to 200. For each cell *c_i_* (*i* = 1,2,…,*m* and *m* is the total number of cells), its expression vector in transcriptomics is denoted as ***te**_i_* = [*te*_*i*,1_,*te*_*i*,2_,…,*te*_*i*,*t*_] and in proteomics as ***pe**_i_* = [*pe*_*i*,1_,*pe*_*i*,2_,…,*pe*_*i*,*p*_], where *t* and *p* are the total number of dimensions in transcriptomics and proteomics, respectively. The Euclidean distances between *c_i_* and *c_j_* are calculated as:


(1) \begin{equation*} d\left(\boldsymbol{t{e}}_i,\boldsymbol{t{e}}_j\right)=\sqrt{\sum_{k=1}^t{\left(t{e}_{i,k}-t{e}_{j,k}\right)}^2} \end{equation*}

(2) \begin{equation*} d\left(\boldsymbol{p{e}}_i,\boldsymbol{p{e}}_j\right)=\sqrt{\sum_{k=1}^p{\left(p{e}_{i,k}-p{e}_{j,k}\right)}^2} \end{equation*}

and the Euclidean distance sets of *c_i_* are obtained as:


(3) \begin{equation*} \boldsymbol{T{D}}_i=\left\{d\left(\boldsymbol{t{e}}_i,\boldsymbol{t{e}}_j\right)|j=1,2,\dots, m\ \mathrm{and}\ i\ne j\right\} \end{equation*}

(4) \begin{equation*} \boldsymbol{P{D}}_i=\left\{d\left(\boldsymbol{p{e}}_i,\boldsymbol{p{e}}_j\right)|j=1,2,\dots, m\ \mathrm{and}\ i\ne j\right\} \end{equation*}

The neighbor sets of *c_i_* are generated as:


(5) \begin{equation*} \boldsymbol{T{N}}_i=\left\{{c}_j|d\left(\boldsymbol{t{e}}_i,\boldsymbol{t{e}}_j\right)\in{\min}_n\left(\boldsymbol{T{D}}_i\right)\right\} \end{equation*}

(6) \begin{equation*} \boldsymbol{P{N}}_i=\left\{{c}_j|d\left(\boldsymbol{p{e}}_i,\boldsymbol{p{e}}_j\right)\in{\min}_n\left(\boldsymbol{P{D}}_i\right)\right\} \end{equation*}

where *n* is the number of neighbors of *c_i_*. The second step is the neighbor pruning. All cells are clustered using the Leiden algorithm [39] based on transcriptome and proteome expression profiles, respectively. The neighbors of *c_i_* are pruned as:


(7) \begin{equation*} \boldsymbol{T{N}}_i^{\prime }=\left\{{c}_j\in \boldsymbol{T{N}}_i| tl\left({c}_j\right)= tl\left({c}_i\right)\right\} \end{equation*}

(8) \begin{equation*} \boldsymbol{P{N}}_i^{\prime }=\left\{{c}_j\in \boldsymbol{P{N}}_i| pl\left({c}_j\right)= pl\left({c}_i\right)\right\} \end{equation*}

where *tl*(*c_i_*) and *pl*(*c_i_*) are the clustering labels of *c_i_* in transcriptomics and proteomics, respectively. The third step is the neighborhood graph construction by neighbor sets of all cells as:


(9) \begin{align*} \boldsymbol{TG}=&\left[\begin{array}{cccc}0& d\left(\boldsymbol{t{e}}_1,\boldsymbol{t{e}}_2\right)& \dots & d\left(\boldsymbol{t{e}}_1,\boldsymbol{t{e}}_m\right)\\{}d\left(\boldsymbol{t{e}}_2,\boldsymbol{t{e}}_1\right)& 0& \dots & d\left(\boldsymbol{t{e}}_i,\boldsymbol{t{e}}_j\right)\\{} \vdots & \vdots & \ddots & \vdots \\{}d\left(\boldsymbol{t{e}}_m,\boldsymbol{t{e}}_1\right)& d\left(\boldsymbol{t{e}}_m,\boldsymbol{t{e}}_2\right)& \dots & 0\end{array}\right]\nonumber\\&\circ \left[\begin{array}{cccc}t{n}_{1,1}& t{n}_{1,2}& \dots & t{n}_{1,m}\\{}t{n}_{2,1}& t{n}_{2,1}& \dots & t{n}_{2,m}\\{} \vdots & \vdots & \ddots & \vdots \\{}t{n}_{m,1}& t{n}_{m,2}& \dots & t{n}_{m,m}\end{array}\right] \end{align*}

(10) \begin{align*} \boldsymbol{PG}=&\left[\begin{array}{cccc}0& d\left(\boldsymbol{p{e}}_1,\boldsymbol{p{e}}_2\right)& \dots & d\left(\boldsymbol{p{e}}_1,\boldsymbol{p{e}}_m\right)\\{}d\left(\boldsymbol{p{e}}_2,\boldsymbol{p{e}}_1\right)& 0& \dots & d\left(\boldsymbol{p{e}}_i,\boldsymbol{p{e}}_j\right)\\ \vdots & \vdots & \ddots & \vdots \\{}d\left(\boldsymbol{p{e}}_m,\boldsymbol{p{e}}_1\right)& d\left(\boldsymbol{p{e}}_m,\boldsymbol{p{e}}_2\right)& \dots & 0\end{array}\right]\nonumber\\&\circ \left[\begin{array}{cccc}p{n}_{1,1}& p{n}_{1,2}& \dots & p{n}_{1,m}\\{}p{n}_{2,1}& p{n}_{2,1}& \dots & p{n}_{2,m}\\{}\vdots & \vdots & \ddots & \vdots \\{}p{n}_{m,1}& p{n}_{m,2}& \dots & p{n}_{m,m}\end{array}\right] \end{align*}

where ***TG*** and ***PG*** are neighborhood graphs in transcriptomics and proteomics, respectively, and


(11) \begin{equation*} t{n}_{i,j}=\left\{\begin{array}{l}1\kern0.5em \mathrm{if}\ {c}_j\in \boldsymbol{T{N}}_i^{\prime}\\{}0\kern0.5em \mathrm{otherwise}\end{array}\right. \end{equation*}

(12) \begin{equation*} p{n}_{i,j}=\left\{\begin{array}{l}1\kern0.5em \mathrm{if}\ {c}_j\in \boldsymbol{P{N}}_i^{\prime}\\{}0\kern0.5em \mathrm{otherwise}\end{array}\right. \end{equation*}

For spatial multi-omics data, the neighborhood graphs are constructed based on spatial coordinates, where each node represents a cell and each edge represents the spatial correlation between two cells. The construction process is similar to that for single-cell multi-omics data, except that the Euclidean distances are calculated using the spatial coordinates of cells. The coordinate data present the distance relationships among cells in real tissues, which helps to construct the neighborhood graphs that are more in line with real scenarios.

#### Dual-path graph attention auto-encoder architecture

The transcriptome expression profile and its neighborhood graph are input into a GATE, and the proteome expression profile and its neighborhood graph are input into another GATE. Each path’s GATE contains an encoder and a decoder. The encoder consists of two graph attention layers [29]. To maintain the capability to focus on important nodes and edges through the graph attention mechanism while also preventing overfitting and conserving computational resources, the attention mechanism is activated in the first layer but deactivated in the second. The decoder adopts a symmetrical structure with the encoder. The “ReLU” and “Tanh” activation functions are used in encoders and decoders for nonlinear transformation.

In each epoch of training, each encoder encodes the inputs into a low-dimensional representation, i.e. embedding. The embeddings from the two paths are integrated as:


(13) \begin{equation*} \boldsymbol{Z}=\left[\boldsymbol{T}_{emb}|\boldsymbol{P}_{emb}\right] \end{equation*}

where ***T**_emb_* and ***P**_emb_* are the transcriptome embedding and proteome embedding, respectively. *Z*, the joint representation, is used to calculate the self-supervision loss. Each decoder reconstructs the corresponding embedding into the original input to calculate the self-reconstruction loss. Self-supervision loss is the core for self-supervised learning, used for multi-omics integration to preserve critical feature information from single omics while ensuring that similar samples maintain their similarity in the joint representation. Self-reconstruction loss is employed to ensure accurate reconstruction of the original input from the joint representation, thereby preserving data integrity and enhancing model robustness. We combine the characteristics of the two losses and adopt a combined weighted loss [34] to effectively train the model as:


(14) \begin{equation*} L=\left({L}_{recon}^t+{L}_{recon}^p\right)+\lambda \left({L}_{spuer}^t+{L}_{spuer}^p\right) \end{equation*}

where *L* is the combined weighted loss, *L^t^_recon_* and *L^p^_recon_* are the transcriptome and proteome self-reconstruction losses, respectively, *L^t^_super_* and *L^p^_super_* are the transcriptome and proteome self-supervision losses, respectively, and *λ* is the balance parameter. The triplet loss function is used to calculate the self-supervision loss, and the mean squared error loss function is used to calculate the self-reconstruction loss. The model parameters are updated through back propagation. When the number of epochs reaches the preset maximum value, the training stops, and the joint representation of multi-omics data, i.e. the integrated embeddings, is finally output.

### Cell clustering and developmental trajectory inference process

The cells are clustered based on the integrated expression profile using the Leiden algorithm [39] to extract the cluster labels. The differentially expressed genes (DEGs) of the cells in each cluster are calculated based on the Wilcoxon test, and those with high confidence are selected as markers based on *p*-values and “log fold change” values. Gene Ontology (GO) enrichment analysis [40] is performed based on these high-confident DEGs to obtain the GO terms corresponding to each cluster, thereby revealing the primary functions of the cells in the cluster. Pseudo-time analysis is conducted based on transcriptome expression profile and cluster labels to infer the developmental trajectory of cells, which helps to understand the developmental process of cells and reveal the diversity and plasticity of cell development.

### Evaluation criteria

For single-cell multi-omics datasets BMNC and SLN111_D1, which provide the ground truth cluster labels of the cells, the integrated data are clustered using the Leiden algorithm [39] to obtain the cluster label of each cell. Then, we calculate Purity (P), Homogeneity Score (HS), Adjusted Rand Index (ARI) and Normalized Mutual Information (NMI) to evaluate different methods. P measures the proportion of cells in each cluster that have the same cluster labels with ground truth, reflecting the accuracy of the clustering results. HS reflects the degree to which each cluster contains only cells from a single category. The ARI measures the consistency between the clustering results and ground truth clusters, taking into account the expected value of random clustering. The NMI measures the mutual information between the clustering results and ground truth clusters. They are common metrics in multi-omics integration studies [18, 19]. To ensure reliable comparison, the “resolution” parameter of the Leiden algorithm is set to 0.1, 0.2, …, 1.0, allowing us to obtain statistical results from 10 sets of independent experiments. We also rank different methods in each metric to calculate Robust Rank Aggregation (RRA), providing a comprehensive evaluation of the performances of different methods [41]. For spatial multi-omics dataset SCS_MT, which has no ground truth, we conduct a series of downstream analyses and visualize the results to demonstrate the facilitative role of SSGATE.

### Project implementation

All scripts are written in Python 3.9. Normalization of transcriptome data, extraction of highly variable genes, calculation and selection of DEGs and proteins, PCA, and clustering based on the Leiden algorithm are implemented through SCANPY (v1.9) [36]. Normalization of proteome data is achieved by replicating the normalization function of Seurat (v5) [16] using Python. GO enrichment analysis is performed using ClusterProfiler4 (v4.0) [42]. Pseudo-time analyses are conducted separately through Monocle 3 (v1.0.0) [43] and Partition-based graph abstraction (PAGA) [44]. The main hyperparameters for SSGATE are set as: maximum epoch value at 300, embedding dimension at 30, number of neighbors at 15, balance parameter at 0.1, and learning rate at 0.001. The project is implemented on STOmics Cloud, utilizing the default computing resources under the “GPU CUDA” node.

## Results

### S‌SGATE’s performance is affected by the number of neighbors and balance parameter

The number of neighbors and balance parameter are the crucial hyperparameters in the neighborhood graph construction and combined weighted loss, respectively. We first fix the balance parameter at 0.1 and verify the effects of the number of neighbors on the performance of SSGATE (Table 2). When the number of neighbors is set to 15, SSGATE achieves optimal results in all metrics on two datasets, particularly excelling in the ARI, where its advantages are most pronounced. We then fix the number of neighbors at 15 and verify the effects of the balance parameter on the performance of SSGATE (Table 3). SSGATE obtains the best results in all metrics on two datasets when the balance parameter is set to 0.1.

**Table 2:** Effects of the number of neighbors on the performance of SSGATE.

Dataset	*n*	P	HS	ARI	NMI
BMNC	10	0.9769 ± 0.0093	0.9372 ± 0.0243	0.4229 ± 0.1119	0.6746 ± 0.0494
	15	**0.9785 ± 0.0078**	**0.9432 ± 0.0217**	**0.4437 ± 0.0946**	**0.6865 ± 0.0442**
	20	0.9702 ± 0.0085	0.9323 ± 0.0228	0.4044 ± 0.0976	0.6665 ± 0.0449
SLN111_D1	10	0.6637 ± 0.0564	0.5960 ± 0.0630	0.4079 ± 0.0230	0.6186 ± 0.0140
	15	**0.6742 ± 0.0502**	**0.6106 ± 0.0549**	**0.4319 ± 0.0507**	**0.6328 ± 0.0140**
	20	0.6715 ± 0.0570	0.6094 ± 0.0626	0.4083 ± 0.0229	0.6270 ± 0.0102

*n* is the number of neighbors.

**Table 3:** Effects of the balance parameter on the performance of SSGATE.

Dataset	*bp*	P	HS	ARI	NMI
BMNC	0.05	0.9784 ± 0.0092	0.9422 ± 0.0250	0.4294 ± 0.0772	0.6808 ± 0.0344
	0.1	**0.9785 ± 0.0078**	**0.9432 ± 0.0217**	**0.4437 ± 0.0946**	**0.6865 ± 0.0442**
	0.3	0.9759 ± 0.0089	0.9310 ± 0.0253	0.3704 ± 0.1008	0.6507 ± 0.0449
	0.5	0.9769 ± 0.0097	0.9331 ± 0.0239	0.3588 ± 0.1153	0.6494 ± 0.0521
SLN111_D1	0.05	0.6681 ± 0.0587	0.6059 ± 0.0625	0.4175 ± 0.0146	0.6292 ± 0.0097
	0.1	**0.6742 ± 0.0502**	**0.6106 ± 0.0549**	**0.4319 ± 0.0507**	**0.6328 ± 0.0140**
	0.3	0.6737 ± 0.0469	0.6018 ± 0.0534	0.3981 ± 0.0331	0.6158 ± 0.0202
	0.5	0.6555 ± 0.0498	0.5838 ± 0.0541	0.3751 ± 0.0297	0.6027 ± 0.0072

*bp* is the balance parameter.

These results indicate that the changes in the number of neighbors and balance parameter can affect the performance of SSGATE. According to the results, we set the number of neighbors at 15 and the balance parameter at 0.1 by default.

### S‌SGATE outperforms competitive methods on single-cell multi-omics integration

**Figure 2:**
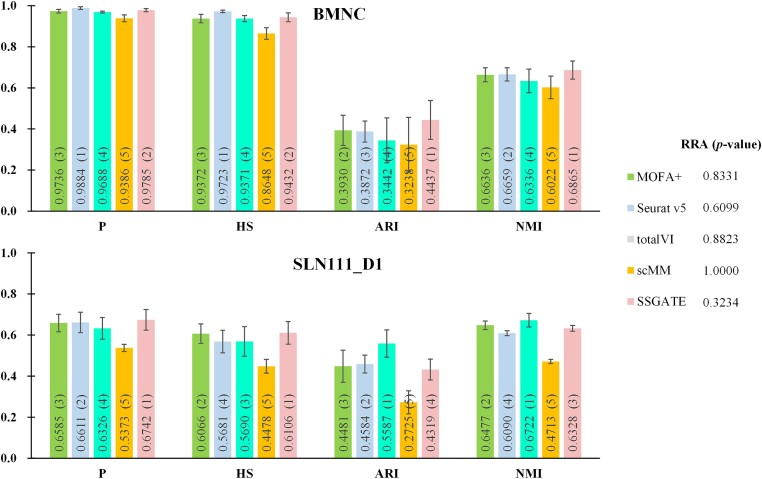
Comparison of SSGATE with competitive methods on single-cell multi-omics datasets. Each result includes the mean and standard deviation values from 10 independent experiments. The numbers in parentheses represent the ranking of that value for the corresponding metric. The smaller the *p*-value of a method’s RRA, the better this method’s overall performance.

SSGATE is compared with four competitive single-cell multi-omics integration methods, including MOFA+ [15], Seurat v5 [16], totalVI [21], and scMM [22], to verify its performance (Fig. 2). TotalVI and scMM are deep learning–based and widely used methods, and MOFA+ (with its latest release on 9 April 2024) and Seurat v5 represent state-of-the-art methods.

On the BMNC dataset, scMM ranks fifth in each metric, with an average ranking of 5. Following is totalVI, which ranks fourth in each metric and has an average ranking of 4. MOFA+ ranks second in ARI and third in the other metrics, giving it an average ranking of 2.75. Seurat v5 ranks first in P and HS, second in NMI, and third in ARI, resulting in an average ranking of 1.75. SSGATE ranks first in ARI and NMI and second in P and HS, with an average ranking of 1.5, making it the highest-ranked method. On the SLN111_D1 dataset, scMM’s average ranking remains at 5. Seurat v5 ranks second in two metrics and fourth in two, with an average ranking of 3. MOFA+ ranks second in two metrics and third in other two, and its average ranking is 2.5. TotalVI, ranks first in two metrics and third or fourth in others, with an average ranking of 2.25. SSGATE ranks first in two metrics, third in one, and fourth in one, leading to the average ranking 2.25, tied with totalVI. Combining the rankings of these methods across all metrics on the two datasets, the *p*-values for RRA are calculated. ScMM has the highest *p*-value, followed by totalVI, then MOFA+, next is Seurat v5, and the smallest *p*-value is obtained by SSGATE. These results indicate that SSGATE outperforms other methods overall.

### S‌SGATE shows strong robustness on single-cell multi-omics integration with noises

**Figure 3:**
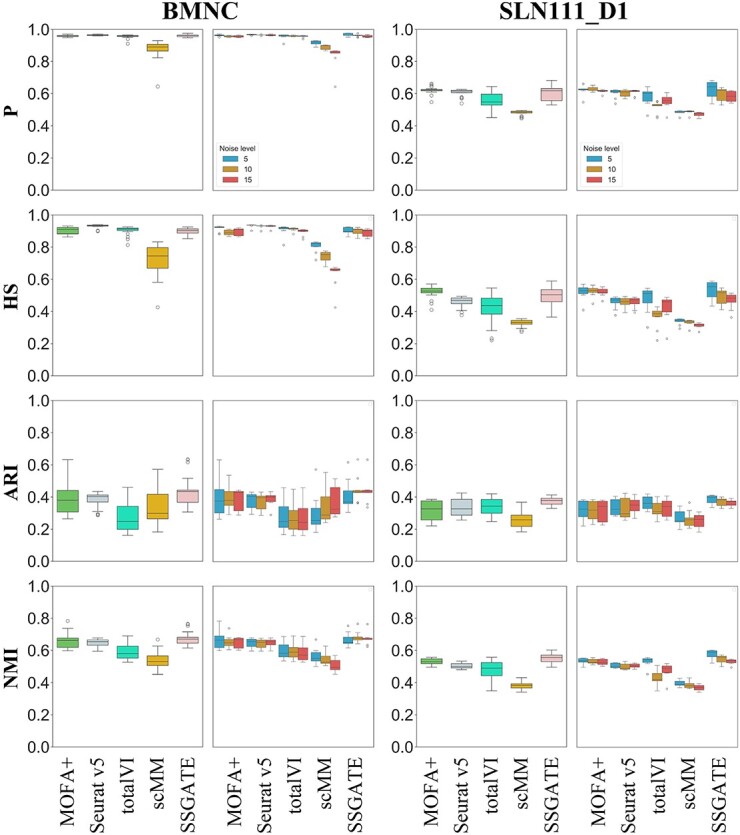
Comparison of SSGATE with competitive methods on single-cell multi-omics datasets with varying levels of Gaussian noise added. On each dataset, the left column is the comprehensive results under three different levels of noise, and the right column is the separate results under three different levels of noise. Noise level 5, 10, and 15 represent 5%, 10%, and 15% Gaussian noise added to the dataset, respectively.

Due to the limitations of existing technologies, the acquired data inevitably contain noise. The manual addition of noise can simulate the situation of poor data quality to verify the robustness of the methods [45]. Therefore, SSGATE is compared with four competitive methods on datasets with varying levels of Gaussian noise added (Fig. 3).

From the separate results, the performances of all methods fluctuate when encountering different levels of noise, with SSGATE’s results being the best in most cases. Notably, scMM’s results exhibit more significant degradation with increasing noise than those of other methods in P and HS on the BMNC dataset, and totalVI’s results obviously fluctuate in P, HS, and NMI on the SLN111_D1 dataset. Comprehensive results under three different levels of noise reveal that on the BMNC dataset, SSGATE achieves the best ARI and NMI, with its P and HS also close to the best results. On the SLN111_D1 dataset, SSGATE still obtains the best ARI and NMI. It also secures the second-best HS and the third-best P. Based on the comprehensive results, scMM, totalVI, Seurat v5, MOFA+, and SSGATE are ranked with average values of 4.88, 3.63, 2.38, 2.25, and 1.88, respectively, with SSGATE being the method with the highest average ranking. Although each method’s results exhibit outliers, which may be due to insufficient stability causing some of the independent experiment results to deviate significantly from other results, SSGATE’s results contain the fewest outliers. For instance, in HS on the SLN111_D1 dataset, SSGATE is the only method with no outliers. These findings indicate that SSGATE can maintain better performance than other methods on the datasets with varying levels of noise, confirming its strong robustness.

### S‌SGATE is applied to spatial multi-omics integration, facilitating cell clustering and developmental trajectory inference

**Figure 4:**
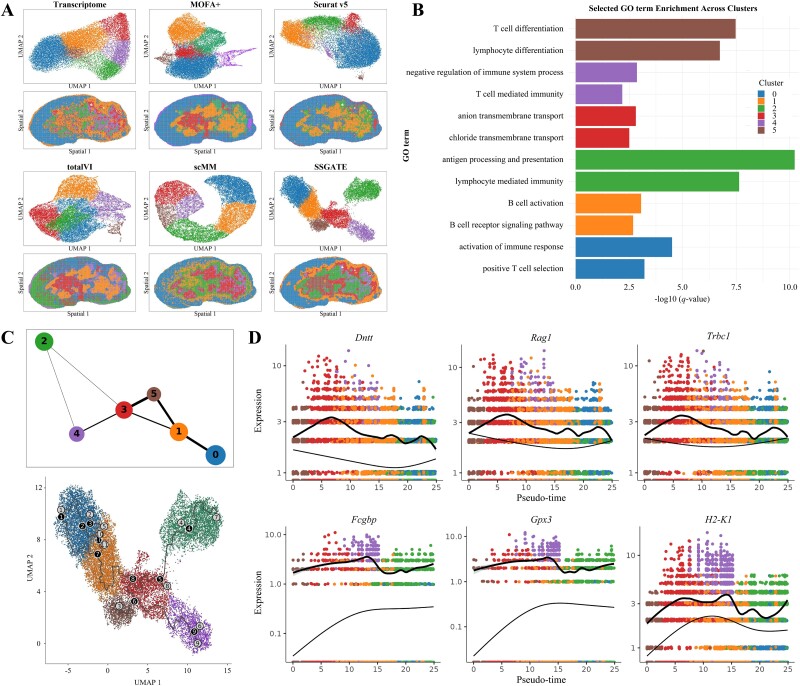
SSGATE is applied to spatial multi-omics integration, facilitating cell clustering and developmental trajectory inference. (**A**) UMAP plots and spatial distribution of the cell clusters for the integrated results of SSGATE and other methods. (**B**) Top two highly enriched GO terms for top 100 ranked differentially expressed genes of the identified cell clusters. (**C**) Upper: PAGA graph for the integrated result of SSGATE, where each node represents a cell cluster, the clusters are connected by weighted edges, the thicker the edge, the stronger the connection. Lower: Monocle 3 trajectory of the cell clusters. (**D**) Pseudo-time kinetics of the significant genes varying along the inferred monocle 3 trajectory. In all subfigures, the cells are colored by the identified cell clusters, as in (**B**).

The spatial multi-omics data from mouse thymus tissue is utilized to demonstrate the benefits of SSGATE for downstream analysis (Fig. 4). From the Uniform Manifold Approximation and Projection (UMAP) plots (Fig. 4A), SSGATE outperforms other methods in learning the integrated and discriminative latent space for tissue section, where cell clusters are more separated from each other. Additionally, the cell clusters from SSGATE’s results display strong spatial aggregation with clear boundaries, which is highly consistent with the fact that the mouse thymus can be broadly divided into the outer cortex region and the inner medulla region [46]. We further demonstrate the effectiveness of SSGATE for combined DEGs and trajectory analysis. The GO-based enrichment analysis of DEGs reveals the progressive maturation, differentiation, and functional specialization of thymocytes within the mouse thymus (Fig. 4B). GO terms associated with thymocyte differentiation and activation are prominently enriched in Clusters 0, 1, and 5, such as T-cell differentiation, B-cell activation, and activation of immune response. Clusters 2 and 4 exhibit GO terms indicative of mature lymphocytes, such as T-cell-mediated immunity and lymphocyte-mediated immunity. In contrast, the significantly enriched GO terms in Cluster 3 are associated with supporting thymocytes development and maturation, such as anion and chloride transmembrane transports. These findings are consistent with the real scenario that thymocytes start to develop in the outer region and migrate toward the inner region [47].

The PAGA graph exhibits a developmental trajectory from outer cortex to inner medulla region, showing high consistency with Monocle 3 results (Fig. 4C). This consistency underscores the reliability of SSGATE in capturing cell development processes. Furthermore, we identify genes that significantly vary over the inferred trajectory. In particular, the genes *Dntt*, *Rag1*, and *Trbc1* are identified to be highly expressed during the early pseudo-time trajectory and then exhibit a gradual decrease. In contrast, the genes *Fcgbp*, *Gpx3*, and *H2-K1* show an increasing trend and reach high expression levels in the later trajectory path (Fig. 4D).

## Discussion

SSGATE achieves the best overall performance on datasets of various tissues from different sequencing technologies primarily due to its ability to construct graph-structured data. Through the GATE framework, SSGATE extracts low-dimensional representations and allows the model to selectively focus on important nodes and edges in the input graph, which is particularly beneficial for extracting significant biological features. Additionally, the use of a combined weighted loss effectively trains the model, enhancing its performance and robustness.

As a self-supervised learning method, SSGATE can perform multi-omics integration without requiring data labels. It adaptively identifies whether the inputs are single-cell or spatially resolved data by retrieving key characters in the data files and then processes them accordingly. Based on experience, we provide a set of hyperparameters suitable for most datasets as default settings, minimizing the need for manual intervention to facilitate usage.

We also record the maximum memory usage of different methods in all experiments. High memory usage may result in method failures due to insufficient computational resources. The maximum memory usage of scMM is 1125 MB, the lowest among the methods, but its overall performance ranking is the lowest among all methods. SSGATE’s maximum memory usage is 3351 MB. Next is totalVI, with a maximum memory usage of 5056 MB. Then comes MOFA+, with a maximum memory usage of 8259 MB. Seurat v5 has the highest memory usage at 12 482 MB, which may be because it typically requires separate normalization, dimensionality reduction, and other operations for each single-omics data before integration, involving a large amount of computation on matrices. Considering both performance and computational resources, SSGATE is the most user-friendly method among these methods.

In cases where the expression profiles of different single-omics can be aligned at the cell dimension, SSGATE has the potential to integrate other single-omics data, in addition to transcriptome and proteome data. Since the expression profiles of different single-omics may vary greatly in feature values ​​and dimensions, the integration of other single-omics data requires reasonable normalization and dimensionality reduction methods, which is also the focus of our subsequent research.

The integration of single-cell multi-omics data with high resolution facilitates more accurate cell classification and identification, while the integration of spatial multi-omics data enables cell heterogeneity exploration and downstream analysis from a spatial perspective [27]. Both types of data have their unique characteristics, and, as a method designed for both single-cell multi-omics and spatial multi-omics data, SSGATE has significant potential for widespread applications.

## Conclusion

This study proposes SSGATE, a multi-omics integration method based on dual-path graph attention auto-encoder for both single-cell and spatially resolved data. SSGATE constructs neighborhood graphs that effectively encapsulate single-cell expression data or spatial information. The dual-path GATE architecture ensures that both shared and modality-specific information are meticulously preserved and utilized, enhancing the comprehensiveness of the integration process. Benchmarking results demonstrate that SSGATE outperforms competitive methods in terms of performance and robustness, and it facilitates downstream analysis. SSGATE provides researchers with a reliable method to extract actionable insights from complex biological data. Future works will focus on optimizing the model’s workflow, improving its efficiency, and exploring its applicability to additional types of omics data.

**Key Points**

- A multi-omics integration method based on dual-path graph attention auto-encoder, named SSGATE, is proposed for both single-cell and spatially resolved data.
- SSGATE can construct the neighborhood graphs based on single-cell expression profiles or spatial coordinates, enabling it to process single-cell data and utilize spatial information from spatially resolved data.
- SSGATE adopts a dual-path graph attention auto-encoder architecture with a combined weighted loss for more effective self-supervised learning.
- SSGATE shows better performance and stronger robustness than competitive methods and facilitates downstream analysis, such as cell clustering and developmental trajectory inference.

## Data Availability

The raw BMNC dataset can be downloaded from National Center for Biotechnology Information (https://www.ncbi.nlm.nih.gov/geo/query/acc.cgi?acc=GSE128639). The raw SLN111_D1 dataset can be downloaded from totalVI repository (https://github.com/YosefLab/totalVI_reproducibility). The raw SCS_MT dataset can be downloaded from Spatial Transcript Omics DataBase (https://db.cngb.org/stomics/project/STT0000094). The format-converted datasets can be found from SSGATE repository (https://github.com/Lv-BioInfo/SSGATE).

## Code availability

The source code of SSGATE is available at https://github.com/Lv-BioInfo/SSGATE.
